# Mumps, Arthritis and Prostatitis

**Published:** 1965-04

**Authors:** G. Ewart Cree


### MUMPS, ARTHRITIS AND PROSTATITIS

BY

G. EWART CREE

Arthritis is an uncommon complication of mumps. Maisondieu (1924) noted six in a series of 1,334 cases, Appelbaum et al (1952) found two in the literature and described a further five. Papers prior to this reported arthralgia only (Wilson 1926, Magida 1951) although in their cases the joints were swollen and inflamed.

Prostatitis in mumps is equally rare. Wesselhoeft (1951) suggest it might be found more often if a routine rectal examination was done; he states that urethral discharge does not occur. Lawrence and McGavin (1948) had no cases of urethritis in their series of 235 cases, but state that it may occur as a complication of mumps.

Gerner (1952) in a report of 179 cases of epidemic mumps noted prostatic involvement in 21 cases. The presenting symptoms were backache and urgency of micturition and examination of the urine showed shreds of mucopus. The prostate was enlarged and tender but this subsided spontaneously in 2–3 days. He states it is a late complication and may occur after the salivary gland swelling has subsided.

### CASE REPORT

A Finnish seaman, aged 28, complained of 14 days pain and enlargement of the left testicle, and urethral discharge for 2 days; also pain in the right knee and left shoulder.

*Examination* showed a well built young man; chest, heart and lungs normal, no generalized or local adenopathy, no skin rash or keratodermia, no balanitis, no conjunctivitis; tongue and mucous membranes normal. The orifice of the left Stenson's duct was more prominent than the right. A mucoid urethral discharge was present, with moderate enlargement of the left testicle and epididymis. Examination of the prostate demonstrated enlargement and tenderness of both lobes. The seminal vesicles were not palpable. The joints showed some pain and stiffness of the left wrist and left shoulder. The right knee was hot and red with a small effusion present. There was ½ in. (1·3 cm) of wasting of the right thigh. A low grade fever was present.

*Investigations.* A urethral smear showed scanty pus cells, some epithelial cells, and a mixed flora; cultures failed to demonstrate gonococci. Urine—slight haze and débris in first glass, clear second glass. Blood serological tests for syphilis negative. Blood count on admission Hb 108 per cent (15·8 g per cent), red cells 4,840,000 million, leucocytes 15,700 per ml. (Polys. 62 per cent, Lymphs. 31 per cent, Mono. 4 per cent, Eos. 3 per cent). B.S.R. 21 mm in the first hour. Urine culture sterile.

The diagnosis appeared to lie between gonorrhoea and an atypical Reiter's syndrome; accordingly treatment was started with Streptomycin 1 gram i.m. twice a day for 3 days, when, through an interpreter, a history was obtained of an acute swelling in the left parotid region 19 days before admission.

Complement Fixation Tests (Virus Reference Laboratory) against mumps antigens showed V. Antigen greater than 1/320, S. Antigen 1/80, suggesting a recent infection with mumps virus.

5 days after admission the B.S.R. had fallen to 4 mm in the first hour, and 6 days later all joints were normal, with the testicular swelling practically subsided.

### DIAGNOSIS

The case against Reiter's syndrome is the absence of keratodermia and ocular lesions, and the presence of orchitis. The rapid resolution of the joint lesion is also unusual in Reiter's syndrome. Against gonorrhoea is the failure to find the organism, and the fact that a testicular swelling appeared before the urethral discharge. The history, course, and laboratory findings make it most probable that in this case arthritis and prostatitis were due to the mumps virus.

*Acknowledgements*

I am indebted to Dr. A. E. Tinkler for permission to publish this record, and to Dr. H. R. Cayton for arranging the serological tests.

### REFERENCES

Appelbaum, E., Kohn, J. L., Steinman, R. E., and Shearn, M. A. (1952). *A.M.A. Arch. int. Med.*, **90**, 217.

Gerner, R. (1952). *Polski Tygodink L.K.*, **7**, 1673.

Lawrence, D., and McGavin, D. (1948). *Brit. M.J.*, **1**, 94.

Magida, M. G. (1951). *Ann. Int. Med.*, **35**, 218.

Maisondieu, P. (1924). Thesis for Doctor of Medicine No. 519. Paris; Jouve. Quoted by Wesselhoeft in Banks H. S. 1951, page 564. Modern Practice in Infectious fevers.

Wesselhoeft, C., in Banks, H. S. (1951). Page 563. Modern Practice in Infectious fevers. Butterworth & Co. ,London.

Wilson, R. E. (1926). *Brit. Med. J.*, **1**, 784.

